# Mangrove Above‐Ground Biomass and Production Are Related to Forest Age at Low Isles, Great Barrier Reef

**DOI:** 10.1002/ece3.72048

**Published:** 2025-09-03

**Authors:** Brooke M. Conroy, Sarah M. Hamylton, Jeffrey J. Kelleway, Emma F. Asbridge, Colin D. Woodroffe, Kerrylee Rogers

**Affiliations:** ^1^ School of Science and Environmental Futures Research Centre University of Wollongong Wollongong New South Wales Australia

**Keywords:** age sequence, blue carbon, coral reef island, drone, LiDAR, mangrove structure

## Abstract

Above‐ground biomass contributes a large proportion of mangrove carbon stock; however, spatio‐temporal dynamics of biomass are poorly understood in carbonate settings of the Southern Hemisphere. This influences the capacity to accurately project the effects of accelerating sea‐level rise on this important carbon store. Here, above‐ground biomass and productivity dynamics were quantified across mangrove age zones dominated by *Rhizophora stylosa*, spanning a tidal gradient atop a reef platform at Low Isles, Great Barrier Reef, Australia. Above‐ground biomass was extrapolated across the forest using field plot data, allometry, a canopy height model derived from remotely piloted aircraft (RPA) LiDAR, and regression analyses. Above‐ground biomass production was calculated as mean annual biomass increments, and canopy production was determined using RPA‐derived multispectral imagery and a Normalized Difference Vegetation Index. Mangrove above‐ground biomass was estimated at 519.7 ± 3.11 t ha^−1^ and increased with age up to the oldest forest (812.0 ± 12.9 t ha^−1^), believed to be ~135 ± 40 years old. Above‐ground biomass was explained by age and tidal position (*r* ^2^ > 0.8), with a positive association between the two predictor variables. Above‐ground biomass production peaked at the lowest intertidal position in the youngest forest aged < 11 years at 36.3 t ha^−1^ yr.^−1^, steadying thereafter, with a mean of 12.5 ± 5.4 t ha^−1^ yr.^−1^ across the island. Production in the canopy remained high until the oldest forest and was negatively associated with age and tidal position (*r* ^2^ > 0.9). Declining production in the older zones corresponded to forest aging, tidal positions becoming suboptimal for growth, and increased exposure to prevailing winds and cyclones. By developing relationships between above‐ground biomass accumulation and age and tidal position, this study informs parameterization of models of the response of biomass to sea‐level rise but requires additional information about relationships between substrate evolution, forest development, and age.

## Graphical abstract

Mangrove above‐ground biomass and productivity was quantified across forest age zones dominated by *Rhizophora stylosa*, spanning a tidal gradient atop a reef platform at Low Isles, Great Barrier Reef. Analysis of historical mapping, field plot data, and RPA‐derived LiDAR data and multispectral imagery indicated above‐ground biomass increased with forest age and tidal position, whereas biomass production peaked in the youngest forest.

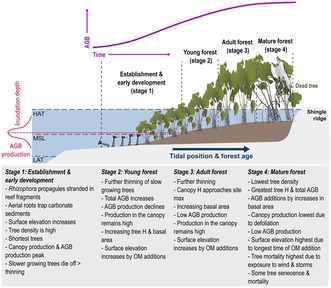

## 1 Introduction

Mangrove structural development is known to influence carbon dynamics through accumulation of biomass (Alongi 2012; Donato et al. 2011; Mitsch et al. 2013), although the specific processes underpinning this relationship vary regionally and are better understood in some settings than others. Mangrove above‐ground biomass (AGB), hereafter referred to as biomass, accumulates over time, with biomass reflecting prior conditions that supported woody biomass addition (Hutchings and Saenger 1987; Saenger 2002). Mangrove biomass and biomass productivity vary spatially at regional and local scales (Rovai et al. 2018; Sanderman et al. 2018), where climate and intertidal position influence mangrove structure and growth (Kauffman et al. 2020; Lamont et al. 2020; Owers et al. 2020; Simard et al. 2019). Spatial patterns of structure, biomass, and productivity in the intertidal zone are closely associated with the underlying geomorphology (Wolanski et al. 1992; Woodroffe 1992), affecting spatial variation in inundation frequency and depth, sediment and nutrient inputs, salinity, and biological processes (i.e., competition for light) (Ball 1998; Saenger and Snedaker 1993). Accordingly, studies quantifying biomass addition across spatial gradients indicate that biomass stocks are related to forest age and stand density (Fromard et al. 1998, 2004; Lamont et al. 2020; Walcker et al. 2018), whereas biomass production is linked to the inundation regime (Clough 1992; Morris et al. 2002; Sherman et al. 2003).

Understanding mangrove structural development with age and accompanying substrate dynamics, particularly in the context of inundation regimes, informs carbon accounting and models predicting the response of mangroves and associated carbon stores under conditions of relative sea‐level rise (Lovelock et al. 2022; Rogers et al. 2019; Walcker et al. 2018). Early studies of mangrove biomass accumulation in Southeast Asian silviculture stands informed harvesting schedules to maximize timber production (Saenger 2002) and exhibit an increase in total biomass with stand age (Aksornkoae 1975; Aksornkoae et al. 1993; Christensen 1978; Ong et al. 1981; Sukardjo and Yamada 1992). However, biomass production varies over time with peak additions at approximately 15–20 years after establishment, and a decline in annual production as mangroves mature and additions are restricted to small increments in the woody constituents (i.e., basal and stem thickening) (Jin‐Eong et al. 1995; Saenger 2002; Alongi 2020). More recently, modelled biomass accumulation in mangrove plantations and natural stands generally show asymptotic behaviour of biomass accumulation with age, indicating a decline in accumulation rates and negligible additions to biomass at maturity, which is estimated between 20 and 40 years of age (Lovelock et al. 2022). Nevertheless, these models indicate substantial variation in biomass accumulation patterns across different geomorphic and climatic regions, underpinning the importance of filling data gaps in poorly represented regions.

Information about biomass dynamics in different geomorphic settings and regions influenced by different Holocene sea‐level histories is required to accurately model the spatial distribution of global mangrove carbon stocks (Rogers et al. 2023). Patterns of mangrove development throughout the Holocene vary globally in relation to postglacial sea‐level changes (Friess et al. 2022; Woodroffe and Davies 2009), suggesting carbon stores may also vary on this basis. There is poor representation of mangrove biomass accumulation patterns and substrate dynamics in carbonate settings which experienced relative sea‐level stability throughout the Holocene, termed far‐field settings (Khan et al. 2015). Low‐wooded islands, where mangroves established on reef platforms under conditions of relatively stable sea levels, represent such settings (Stoddart 1980; Woodroffe 1987). Although little is known of mangrove carbon accumulation on the low‐wooded islands of the Great Barrier Reef, reef island mangroves can be as productive as estuarine settings (Woodroffe 1987), and given adequate conditions, mangrove lateral expansion and biomass accumulation has occurred over the last hundred years in these settings (Hamylton et al. 2019, 2023).

Remote sensing techniques have been widely used to assess mangrove ecological functions and/or health (Canty et al. 2025; Ju et al. 2025; Pham et al. 2019). Of relevance for mangrove carbon stock assessments, highly accurate elevation information from Light Detection and Ranging (LiDAR) can characterize mangrove tree height (Fatoyinbo et al. 2018; Feliciano et al. 2017). These data can be leveraged to quantify AGB over large spatial scales on the basis of relationships between tree height and AGB (Salum et al. 2021; Wong et al. 2024). Remotely piloted aircraft (RPA) fitted with LiDAR sensors are being increasingly used to quantify mangrove structure, functioning, and biomass (Guo et al. 2021; Wang et al. 2020), particularly for studies requiring data of high spatial and/or temporal resolution, and in locations where LiDAR data are not available. Accurate canopy height models (CHMs) from LiDAR in combination with previously developed allometric equations, relating vegetation structure to mass, can be used to quantify AGB at broader scales and in different locations (Chave et al. 2005; Komiyama et al. 2008; Salum et al. 2021). Greatest accuracy of biomass estimates is attained when applying species‐ and region‐specific allometric equations that capture variability of mangrove structures across species and climatic and geomorphic settings (Estrada and Soares 2017; Owers et al. 2018). Moreover, scaling biomass from the plot scale to larger scales using remote sensing maintains accuracy of biomass estimates (Salum et al. 2020, 2021).

Quantification of mangrove production reveals processes influencing biomass and carbon stores; however, total mangrove production is difficult to assess, and studies typically measure only one aspect of production, such as leaf, root or biomass production (Hutchings and Saenger 1987). Reliable estimates of mangrove production are derived by time‐averaging increases in AGB, represented as mean annual increments over the age of a stand (Saenger 2002; Alongi 2002). Alternatively, the relative ease of monitoring mangrove canopies using optical imagery has encouraged the application of remotely sensed indices such as the Normalized Difference Vegetation Index (NDVI), leveraging the red and near‐infrared bands of multispectral imagery which are sensitive to chlorophyll and can thereby monitor leaf area and photosynthetic production (Guo et al. 2021; Pastor‐Guzman et al. 2018). Nevertheless, there are few remote sensing studies of mangrove productivity, despite the recent opportunities provided by advances in technology.

This study seeks to address knowledge gaps regarding mangrove forest structural development, biomass, and production in coral reef settings of the Southern Hemisphere. Low Isles is a low‐wooded island and has been extensively studied since it was mapped during the 1928–29 Great Barrier Reef Expedition (Spender 1930; Yonge 1930), offering a unique opportunity to expand on this knowledge of reef island mangrove development. This site is relatively undisturbed, being situated in the Great Barrier Reef World Heritage Area, facilitating observation of the natural dynamics of mangrove forest development. The aim of this study is to establish relationships between AGB, production, forest age, and tidal position at Low Isles, Great Barrier Reef, Australia. The specific objectives of this study are to:

1. Establish a relationship between mangrove AGB and tree height using field plot measurements and allometry.
2. Extrapolate AGB across the mangrove forest using this relationship and an RPA‐LiDAR‐derived CHM.
3. Explore relationships between mangrove AGB, productivity, tidal position, and forest age.

Characterizing spatial and temporal variation in mangrove structural development and biomass accumulation in different geomorphic settings facilitates accurate estimations of carbon sequestration variation with forest age and across different settings. This study both contributes to the improvement of global models that parameterize mangrove biomass and serves as the first comprehensive report on biomass accumulation for a low‐wooded island of the Southern Hemisphere.

## 2 Methods

### 2.1 Study Site

**FIGURE 1:**
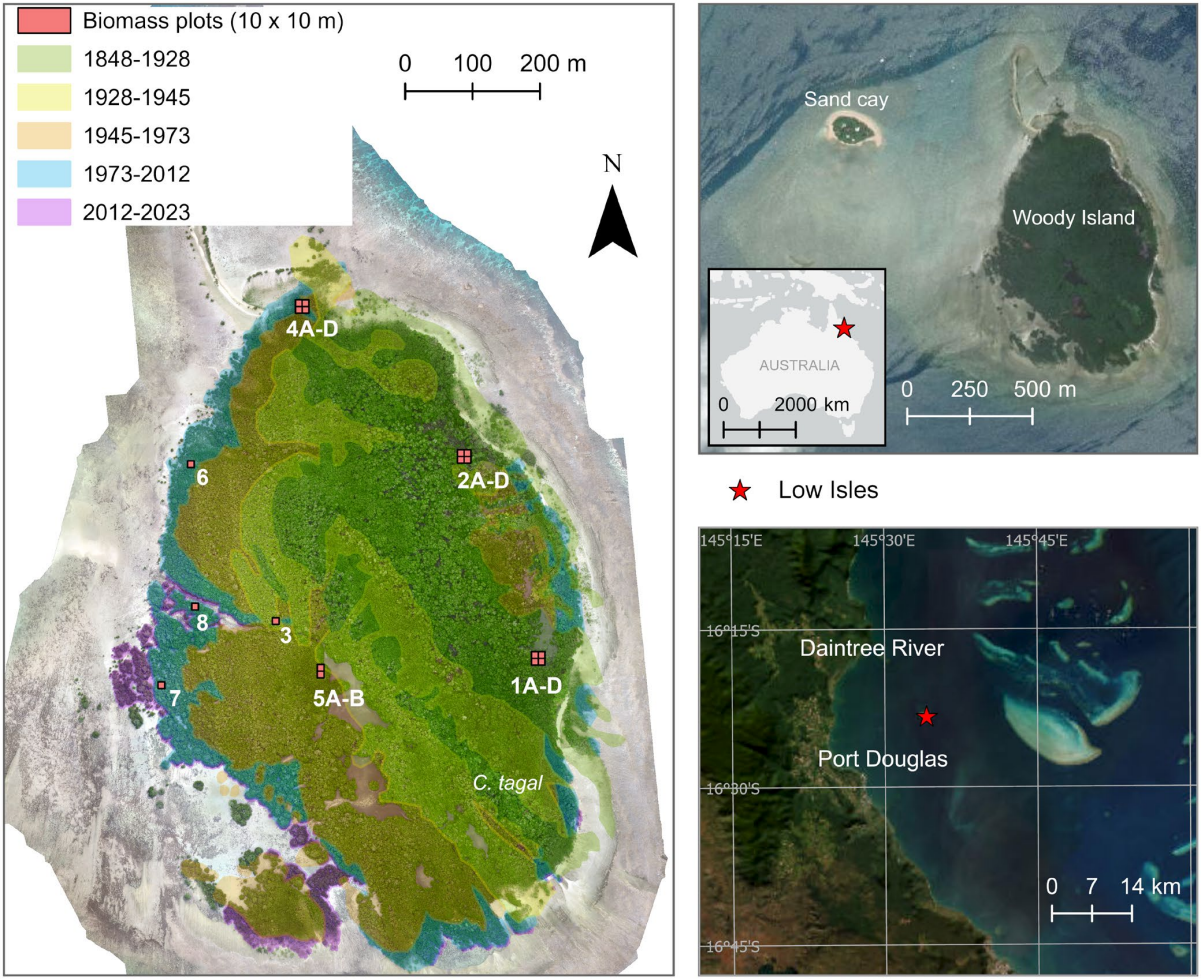
Study site and location of biomass plots. The orthomosaic was generated from an RPA survey in 2023 (DJI Matrice 300 RTK) and the forest age zone extents are overlaid. Photographs of field plots are included in Figures A1 and A2.

Low Isles is the southernmost low‐wooded island on the Great Barrier Reef, Australia, located on the inner shelf ~40 km west of the outer barrier of the Great Barrier Reef (16°23′ S, 145°34′ E) (Figure 1). Low Isles includes a sand cay and adjoining mangrove forest named Woody Island atop a distinctive horseshoe‐shaped reef platform. The entire island, including the mangrove forest, is referred to as Low Isles for simplicity in this study.

**FIGURE A1:**
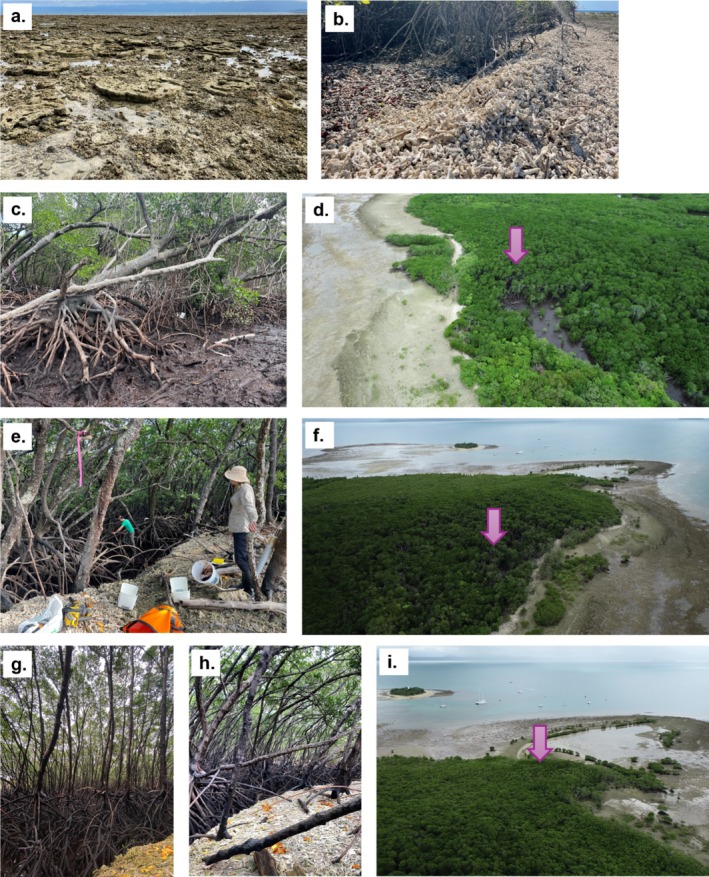
Photographs taken in the field on Low Isles in 2023. Reef flat and microatolls (a), shingle ridge on the eastern boundary of the mangrove forest (b), plot 1 mangroves (c), plot 1 RPA image (d), plot 2 mangroves (2), plot 2 RPA image (f), plot 4 mangroves (g and h) and plot 4 RPA image (i).

Low Isles has a semidiurnal tidal regime and a high tidal range of up to 3.8 m. Sediments are primarily carbonate sands and gravels and are transported across the reef platform in a northwesterly direction by southeast swells, with the prevailing current inhibiting dispersal of terrigenous sediments across the shelf (Hopley 1982; Larcombe and Carter 2004; Larcombe and Woolfe 1999). Southeast trade winds dominate for ~9 months of the year, and a northwest monsoon occurs in the summer months (December–February), causing heavy rainfall and occasional cyclones from the Coral Sea where storm surges > 1 m (for cyclones with a 10‐year return period) and waves up to 5–7 m can occur (Hopley 1982). Mean annual rainfall recorded from the rain gauge at Low Isles between 1982 and 2024 exceeded 2000 mm (Bureau of Meteorology 2024). Cyclones frequent the region (Frank and Jell 2006), and changes to the ramparts, shingle ridges, and mangrove forest at Low Isles were attributed to strong winds, large waves, and higher tidal waters associated with severe cyclones in 1931, 1934 and 1950 (Moorhouse 1936; Stephenson et al. 1958). The rampart is a partially lithified conglomerate comprising shingle material (i.e., dead coral fragments and branches), bounding the mangrove forest on the windward, eastern side of the island (Spender 1930). Unconsolidated shingle material accumulates on the ramparts (see Figure A1b) and in some areas rises from the mangrove mud by up to 2 m at a 60° angle (Hamylton et al. 2019). These structures form a protective boundary for mangroves from southeast trade winds, enabling leeward expansion and seedling development by providing mechanical support (Spender 1930).

The first European mention of Low Isles was by Cook in 1770 (Beaglehole 1955, 343), followed by King et al. in 1819 (King et al. 1827, 207) and MacGillivray in 1848, who mentioned, “…mangroves on the reef, the roots of which are washed at high water…” (MacGillivray et al. 1852, 101–103). An extensive survey of the sand cay, reef platform and mangrove at Low Isles was undertaken by members of the 1928–29 Great Barrier Reef Expedition (Yonge 1930). A detailed 1:5000 map from this expedition was constructed by combining theodolite triangulation with aerial photography (September of 1928), characterizing in detail, the reef platform morphology and mangrove forest extent (Spender 1930; Steers 1929). This work mapped a dense *Rhizophora* woodland named the “mangrove swamp” with features such as sandy pools, muddy glades, and passages and shingle tongues extending inwards from the ramparts and is represented by the 1928 extent in the present study. An open “mangrove park” comprising *Rhizophora* saplings and outlying trees was mapped to the southwest of the mangrove swamp (Stephenson et al. 1931), but was not included in the 1928 extent.

A subsequent map survey by tracing aerial photographs in January of 1945 documented an absence of marked change in the extent of the mangrove swamp but reported development of *Rhizophora* in the mangrove park where trees were expanding and coalescing (Fairbridge and Teichert 1948). Further mapping by tape‐and‐compass traverse in 1973 documented significant mangrove extension and a conversion to continuous woodland in the mangrove park, though trees were shorter and less dense than those of the 1928 swamp, which remained largely unchanged (Stoddart et al. 1978). These studies also observed mangroves on the ramparts including species such as *Avicennia marina* var. *eucalyptus, Bruguiera gymnorhiza, Aegialitis annulata, Aegiceras corniculatum, Ceriops tagal, Excoecaria agallocha*, and *Osbornia octodonta*. *Rhizophora stylosa* is the dominant mangrove species and forms almost the entirety of the continuous forest on the reef platform, mostly as closed forest, and was reported up to 20 m tall in previous work (Stoddart et al. 1978). Seedlings > 1 m tall were attributed to the large tidal range (Stoddart et al. 1978).

The analysis in this study is focused on the *R. stylosa‐*dominant forest, estimated to comprise > 95% of the mangrove cover. Field expeditions in the present study were conducted in June 2022 to collect RPA‐derived multispectral imagery and in July 2023 to collect field plot data and RPA‐derived LiDAR data. A patch of *C. tagal* was identified in field expeditions in 2023 on an interior ridge towards the southeastern corner of the mangrove forest, consistent with the 1928 rampart and labelled on the orthomosaic (Figure 1). On the leeward side of the reef platform, *R. stylosa* expanded in a westerly direction over the past ~90 years (Hamylton et al. 2019). Detailed information about the reef and sand cay morphology and change over time is provided in Hamylton et al. (2019).

### 2.2 Field Plot Tree Structure, Density, and Biomass

**FIGURE 2:**
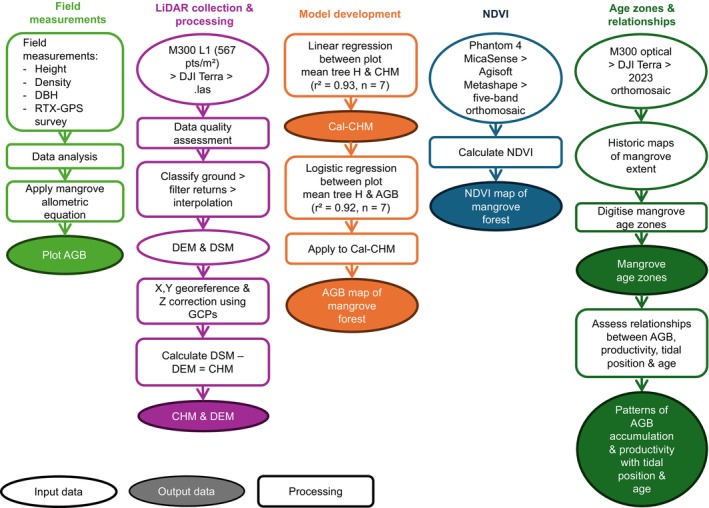
Workflow for the collection and processing of field‐based and remote sensing datasets.

**FIGURE A2:**
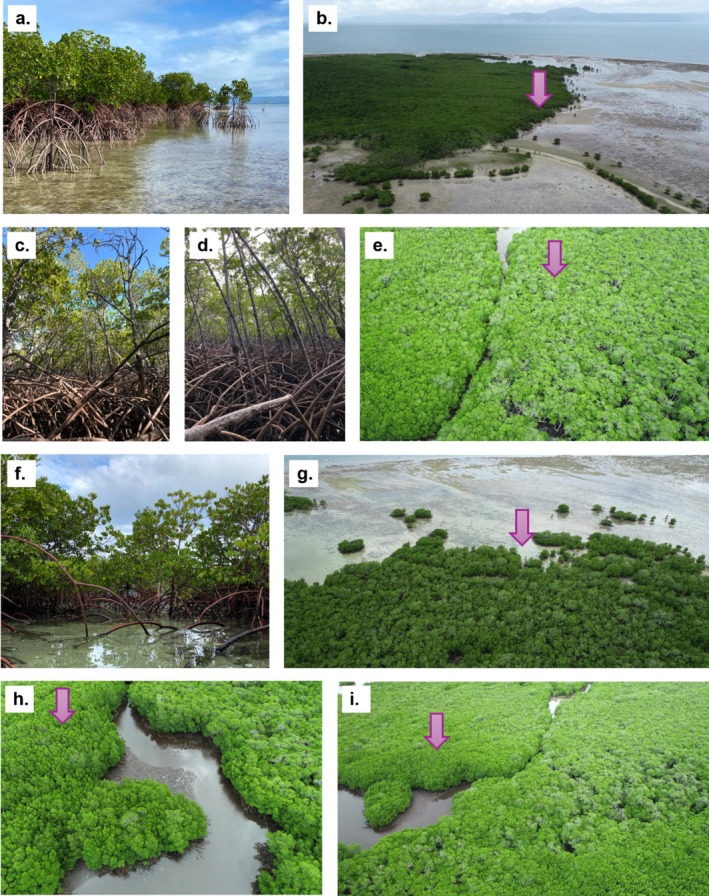
Photographs taken in the field on Low Isles in 2023. Plot 6 mangroves (a), plot 6 RPA image (b), plot 3 mangroves (c and d), plot 3 RPA image (e), plot 7 mangroves (f), plot 7 RPA image (g), plot 5 RPA images (h and i). Note that no images are available for plot 8 but the structure was similar to plot 6 and 7.

A total of 18 field plots (10 × 10 m) were established in the *R. stylosa‐*dominant forest to collect tree measurements. Plots were positioned to span mangrove age zones and varying forest structures, assessed using historic mapping and field observations, respectively. Plots 1A–D and 2A–D are positioned in the 1848–1928 forest, and plot 3 is in the 1928–1945 forest but close to the margin of the 1945–1973 forest. Plots 4A–D are in the 1945–1973 forest but also overlap with the 1973–2012 forest. Plots 5A and 5B are in the 1945–1973 forest, and plots 6, 7, and 8 are in the 1973–2012 forest. The four nested plots for 1A–D, 2A–D, and 4A–D were sampled because of field observations of lower tree density that may require pooling the data from each subplot to calculate biomass for the larger 20 × 20 m plot. To determine if subplot pooling is required, significant differences in tree structure (i.e., trunk diameter at breast height, DBH) between the subplots for 1A–D, 2A–D, and 4A–D were tested using a one‐way ANOVA and Tukey Honestly Significant Difference (HSD) test. ANOVA tests throughout this manuscript were tested under the assumptions of normality and homogeneity of variances. Significance for ANOVA and Tukey HSD tests was indicated by an alpha level of < 0.05, demonstrating no significant difference between subplots and subplot pairs, justifying treating subplots separately (Table A1). Photographs of field plots are provided in Figures A1 and A2. The workflow for data collection and processing is provided in Figure 2.

Individual tree AGB in field plots was estimated using an allometric equation specific to both the species and region that uses the trunk DBH, a tree metric that is related to AGB (Clough and Scott 1989). A region‐ and species‐specific allometric equation was prioritized to improve accuracy by accounting for the strong influence of climatic variables and species structure on tree AGB (Komiyama et al. 2008), compared to general allometric equations, which may use both tree DBH and height. Additionally, resource and time constraints in the field meant not all tree heights were measured; therefore, to calculate AGB for each plot, a relationship using DBH only was necessary.

To calculate field plot AGB, tree trunk circumference at breast height (at 1.3 m) was measured and converted to DBH (cm). If trees were multi‐stemmed, each stem was treated as a separate tree. For leaning trees, DBH was measured at 1.3 m along the natural height of the trunk or above the highest prop root when this arose from the stem at a height above 1.3 m. This approach is consistent with the development of allometric equations from DBH. Individual tree heights were recorded in seven field plots. However, because of time constraints, one tree height was measured in plots 2A–D; a maximum tree height was measured in plots 1A–D, and tree height data were not collected for the remaining five plots. Tree density was recorded as the number of individuals included in AGB calculations (i.e., above 1.3 m) and trees below this height were also tallied to determine shrub density. A total of 825 trees were measured for DBH, and a total of 641 trees were measured for tree height (see Table A2 for the number of measurements per plot). AGB for each tree was determined using Equation (1), an allometric equation on the basis of DBH (Clough and Scott 1989):

(1) $$\mathrm{AGB}\;\left(\mathrm{kg}\right)=10^{A+B\times \log_{10^{\mathrm{DBH}}}}$$

where 10 represents the antilog of a base 10 log from rearranging the equation from (log Biomass = *A* + *B* log DBH) (Clough and Scott 1989). The equation coefficients were derived from the total AGB of both *R. stylosa* and *Rhizophora apiculata* in northeastern Queensland (Clough and Scott 1989), where *A* is −0.9789 and *B* is 2.6848. This equation was developed from trees with a DBH range of 3–23 cm, and therefore, there is some uncertainty in values outside this range.

Individual tree AGB was totalled to determine the total plot AGB and was divided by the plot size (0.1 ha) to determine AGB per unit area (t ha^−1^). To assess the suitability of an allometric equation using only DBH, a linear regression was generated between plot mean tree height and mean tree DBH. To explore the interaction between plot tree density and DBH, both linear and logistic regressions were generated. The coefficient of determination (*r* ^2^) was used to indicate the suitability of regressions.

### 2.3 Remote Sensing Data Collection and Processing

RPA remote sensing was used in this study as high‐density LiDAR and high‐resolution optical imagery were required, and there was no other airborne LiDAR data available. An RPA survey of the mangrove forest was conducted in July 2023 using a DJI Matrice 300 RTK, fitted with a Zenmuse L1 and optical sensor to collect LiDAR data and imagery in the visible spectrum (red, green and blue), respectively. Multispectral imagery (five‐band) was collected in June 2022 using a DJI Phantom 4 fitted with a MicaSense RedEdge‐M sensor. RPA surveys were conducted at low tides, where possible, to optimize detection of the substrate surface using LiDAR and spectral bands, both of which are sensitive to water (Klemas 2013). Given the density of mangrove at this site, RPA‐based LiDAR sensing was identified as the optimal approach for developing digital elevation models (DEMs), digital surface models (DSMs), and CHMs because of its high spatial resolution and precision. RPA survey details are provided in Table A3.

Prior to the RPA survey, seven ground control points (GCPs) were placed across a range of elevations on the reef platform and within the mangrove forest and were surveyed using a Trimble Real Time Extended Global Positioning System (RTX‐GPS). RTX‐GPS points were also collected for easily identifiable features in the imagery, such as microatolls and small mangroves on the reef platform. A total of 12 GCPs were surveyed using RTX‐GPS (vertical error of 0.197 cm) to correct the vertical and horizontal offset of rasters.

Initial processing of the RPA‐derived LiDAR data and the 2023 RPA‐derived imagery was conducted in DJI Terra (v.3.9.4), generating a LAS file and orthomosaic (2.78 cm/pixel), respectively. A multispectral orthomosaic (8 cm/pixel) was created by stitching images for each of the five spectral bands (blue, green, red, red edge, and near‐infrared) in Agisoft Metashape Professional (Metashape v.1.6.3). Further image analysis was performed using ArcGIS Pro (v.3.3.1; ESRI) and R Studio (v.4.3.3; R Core Team 2024).

LiDAR ground points were classified to generate a DEM and DSM at 0.25 m resolution (Table A4). The visible spectrum orthomosaic, DEM and DSM were corrected for horizontal offset by georeferencing using the GCPs and a first‐order polynomial correction (RMS errors < 3 cm). A reliable RTX‐GPS point was not available for the DJI Matrice 300 RTK base station at the time of the survey, which created a systematic vertical offset of the digital models. To rectify this, the average vertical offset between the DEM and GCPs was subtracted from both the DEM and DSM. The GCPs used for vertical correction were filtered to > 0.28 m relative to the Australian Height Datum (AHD) to ensure submerged features did not influence the correction. This threshold was selected upon inspecting the orthomosaic for submerged features, as LiDAR returns do not effectively penetrate water.

### 2.4 Mangrove Forest Age Zones

The boundary of the *R. stylosa‐*dominant forest in 2023 was digitized using the visible spectrum orthomosaic. Mangroves on the reef flat and shingle ridge not attached to the continuous forest were excluded from the 2023 extent to limit inclusion of the patchy extent of other mangrove species and to improve the LiDAR‐derived AGB model. Mangrove forest age zones prior to 2023 were visually digitized using previously mapped extents and include the 1928, 1945 and 1973 extents (Fairbridge and Teichert 1948; Spender 1930; Stoddart et al. 1978). An aerial image from 2012 was digitized to create a 2012 mangrove extent. Additional shapefiles were created to define age zone boundaries and include 1848–1928, 1928–1945, 1945–1973, 1973–2012, and 2012–2023. A lower limit of 1848 for the oldest forest was determined on the basis of observations of mangroves at Low Isles in 1848.

### 2.5 Mangrove Structure, Biomass, and Greenness

Mangrove structure was quantified using a CHM (pixel size: 0.25 m) by subtracting the DEM from the DSM. The CHM was clipped to the 2023 mangrove extent and was resampled to a pixel size of 10 m (100 m^2^) to align with the field plot resolution for AGB. Field notes and GPS points guided the location and construction of square polygons representing the biomass plots (10 × 10 m). Calibration of the CHM was performed by linear regression analysis between the field plot mean tree height, where all tree heights were measured (*n* = 7), and the corresponding CHM pixel value to improve the accuracy of the models. The calibrated CHM is hereafter referred to as Cal‐CHM.

AGB was extrapolated across the mangrove forest using the Cal‐CHM and a logistic regression relating field plot mean tree height and total plot AGB (*n* = 7) (Equation 2). As mangrove tree height asymptotes at a maximum height (Suwa et al. 2008), a logistic regression was most appropriate to explain the data. In some cases, linear regressions exhibit greater capacity to describe relationships between mangrove AGB and height (Suwa et al. 2008). However, they cannot effectively describe the AGB addition of very large trees that exhibit an asymptote in growth with age.

(2) $$Y=\frac{c}{1+e^{-a\times \left(x-b\right)}}$$

where *a* is the growth rate estimated at 0.323, *b* is the inflection point estimated at 11.78 m, and *c* is the asymptote estimated at 31.77 t per plot. The field plot mean tree height was calculated for the seven plots with tree height data for all trees and is represented by *x*.

To assess mangrove canopy greenness, the red and near‐infrared multispectral orthomosaics were used to calculate NDVI. These bands detect the absorbance of chlorophyll and the reflection of the mesophyll (Pettorelli et al. 2005). NDVI is correlated with canopy closure in mangroves (Jensen et al. 1991), providing a proxy for canopy greenness and leaf area, indicating production of the canopy (Carlson and Ripley 1997; Clough, Ong, and Gong 1997). However, spectral bands used in NDVI are sensitive to water, and therefore, the influence of tides, substrates and exposed roots will influence NDVI when the canopy is sparse (Rogers et al. 2017). The NDVI raster was georeferenced to correct horizontal offset using the visible spectrum orthomosaic. Obvious features were co‐located in both rasters with a total of 11 control points used to correct the NDVI raster using a first‐order polynomial correction (RMS errors ~30 cm).

The Cal‐CHM, AGB, DEM, and NDVI rasters were resampled to 10 m resolution, clipped to the 2023 mangrove extent and interior ponds were removed to reduce outliers resulting from poor accuracy of LiDAR points and multispectral imagery in submerged features. Values reported in the results represent the summary statistics of 10 m pixels.

### 2.6 Mangrove Productivity

In this study, two measures of mangrove production were estimated, one using NDVI and the other using the AGB model. The NDVI rate represents production in the canopy by estimating the trajectory of leaf area cover in the upper canopy and was calculated as the rate of NDVI change between adjacent age zones using Equation (3):

(3) $$\text{NDVI production rate}\;\left({\mathrm{yr}}^{-1}\right)=\frac{A_{1}-A_{2}}{{\text{NDVI}}_{1}-{\text{NDVI}}_{2}}$$

where *A*_1_ and *A*_2_ represent the mid‐point of adjacent age zones, and NDVI_1_ and NDVI_2_ represent the mean NDVI pixel values for each adjacent age zone.

Total mangrove above‐ground productivity was calculated using the AGB model and represents the annual biomass increment addition, calculated for each age zone by dividing AGB by age. AGB represents the sum of biomass in each age zone, and age represents the forest age in years by subtracting the mid‐point of each respective age zone from 2023 (i.e., current forest extent). Total above‐ground productivity focuses on the additions to total biomass and is considered a more accurate measure of production, incorporating the larger components of biomass addition. In contrast, NDVI focuses on production in the canopy by assessing changes in greenness and is unlikely to be representative of biomass production that contributes to carbon storage.

### 2.7 Relationships Between Mangrove Biomass, Productivity, Tidal Position, and Age

The distribution of raster values within the forest age zones was visualized using violin plots overlaid with boxplots for the Cal‐CHM, AGB, DEM, and NDVI rasters. Surface elevation determined using the DEM (m AHD) was used as a proxy for tidal position at the site, and values represented the mean DEM values within each age zone.

To assess whether there was a significant difference in mangrove structure, biomass, tidal position, and greenness across age zones, a one‐way ANOVA was conducted for each raster (i.e., CHM, AGB, DEM, and NDVI). A Tukey HSD test was conducted to test for significant differences in rasters between each age zone.

Relationships between AGB and forest age were assessed by performing linear and logistic regressions using the sum of AGB in each age zone from the raster divided by the area of the respective age zone. This analysis was repeated for AGB and tidal position. Relationships between mangrove production, forest age, and tidal position were explored using the NDVI and AGB production data for each age zone. Linear regressions were generated where appropriate, but given the small number of data points (*n* = 5), complex non‐linear relationships were not explored for production. Scatter plots were used to visualize relationships between AGB production across age zones and tidal position.

To determine whether the variation in AGB was explained by forest age and/or tidal position, a mixed‐model ANOVA was conducted using all raster values associated with each age zone (i.e., AGB and DEM). The significance of fixed effects was reported to ascertain whether forest age and tidal position independently explained variation in AGB. If the interaction effect (age zone*tidal position) was significant, this suggests that the effect of forest age zone on AGB depends on tidal position. Statistical analyses were conducted in R Studio (v.4.3.3; R Core Team 2024) and JMP (JMP Pro 17; SAS Institute Inc., Cary, NC, USA).

## 3 Results

### 3.1 Field Plot Tree Structure, Density, and Biomass

**FIGURE 3:**
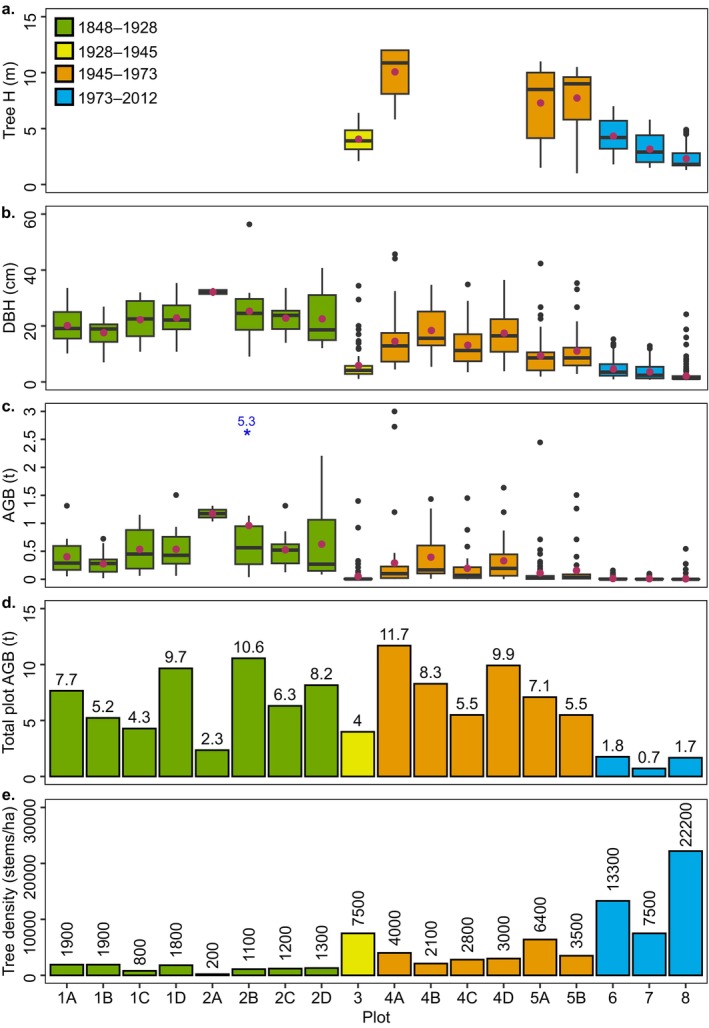
Field plot boxplots for tree height (a), DBH (b), and AGB (c) and bar charts of total AGB (d) and tree density (e). Plot age generally decreases from left to right. Horizontal line inside boxplots indicates median, maroon dot represents the mean, the box indicates 25th and 75th percentiles and whiskers indicate maxima and minima. Blue asterisks and label on AGB boxplot (d) represent outliers beyond the plot scale. Field plot data is provided in Table A4.

Field plot tree height ranged between 1.3 and 12.0 m with taller trees located in the older plots (Figure 3a). The distribution of DBH and AGB per tree across the field plots shows a general pattern of greater DBH and AGB in older plots. DBH ranged between 0.6 and 56.3 cm, and AGB ranged between 0.00003 and 5.27 t (Figure 3b,c). Total plot AGB converts to AGB per area ranging between 71 and 1170 t ha^−1^ with a mean ± SE of 612 ± 75 t ha^−1^ (Figure 3d). Field plot tree density was generally lower in the older plots, where the mean tree density ± SD of trees > 1.3 m tall in plots aged between 1848 and 1945 was 1967 ± 2025 stems ha^−1^, compared to 7200 ± 6235 stems ha^−1^ for plots aged between 1945 and 2012 (Figure 3e, Table A5).

**FIGURE 4:**
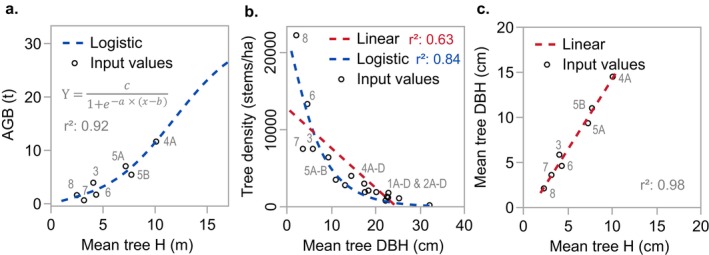
Field plot tree metric relationships including a logistic regression between mean tree height and total AGB (*n* = 7) (a), linear and logistic regression between mean tree DBH and tree density (*n* = 18) (b) and linear regression between mean tree height and mean tree DBH (*n* = 7) (c). Logistic regression parameters for (a) are provided in Table A6. Plot names are labeled. Standard error is not included as it is within the margin of data points.

The relationship between plot mean tree height and total AGB was explained by a logistic regression (*r* ^2^ = 0.92, Figure 4a) and is considered reliable up to the maximum field plot mean tree height of 10.1 m but was not tested beyond this height using field data. Caution is advised when using this equation to predict AGB for tree heights > 10 m; however, only 10.5% of the data were above this height (Figure A4). The relationship between mean tree DBH and tree density was best explained by a logistic regression and shows increasing DBH with declining tree density, with the older plots having the lowest tree density (*r* ^2^ = 0.84, Figure 4b). A positive linear regression explained the relationship between mean tree height and mean tree DBH (*r* ^2^ = 0.98, Figure 4c).

### 3.2 LiDAR Model Calibration and Validation

**FIGURE A3:**
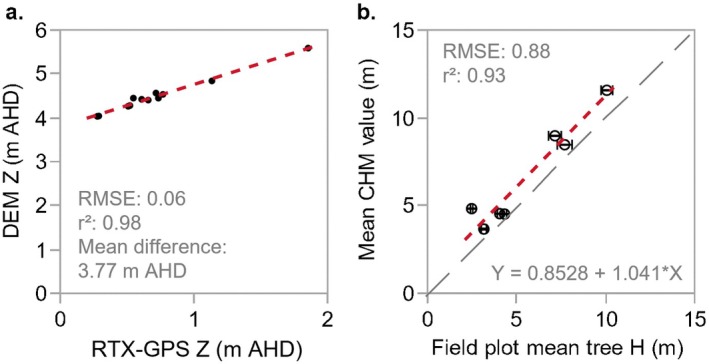
Calibration of remote sensing data using linear regression between the RTX‐GPS Z (m AHD) and the DEM Z (m AHD) (0.25 m resolution) with the difference of the reliable points (points > 0.28 m AHD) used to calibrate the DEM and DSM (*n* = 12) (a). Linear regression comparing plot mean tree height to the mean CHM pixel value within the plot boundaries with error bars representing the standard error of the mean (*n* = 7) (b).

**FIGURE A4:**
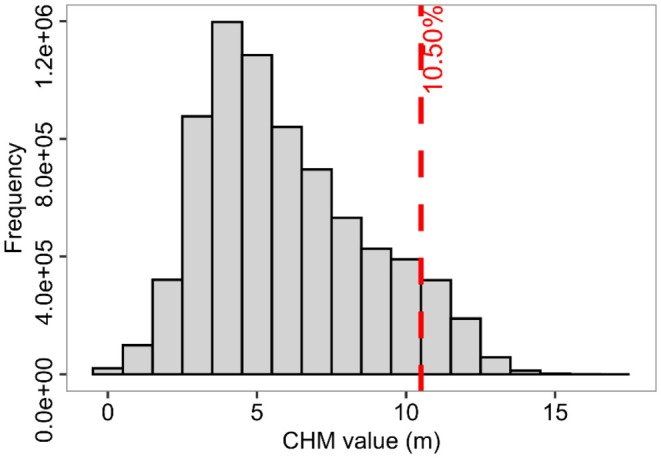
Histogram of CHM values with a red dashed line indicating the maximum field plot mean tree H used to develop the logistic model. 10.5% of raster values are outside the model range.

The vertical DEM offset was corrected using the mean difference between the RTX‐GPS and DEM vertical position from the linear regression (*r* ^2^ = 0.98, RMSE = 0.06) (Figure A3a). Calibration of the CHM was performed using the equation from the linear regression between field plot mean tree height and the mean CHM value (0.25 m resolution) within the plot boundaries (*r* ^2^ = 0.93, RMSE = 0.88 m) (Figure A3b).

### 3.3 Relationships Between Mangrove Biomass, Productivity, Tidal Position, and Age

**FIGURE 5:**
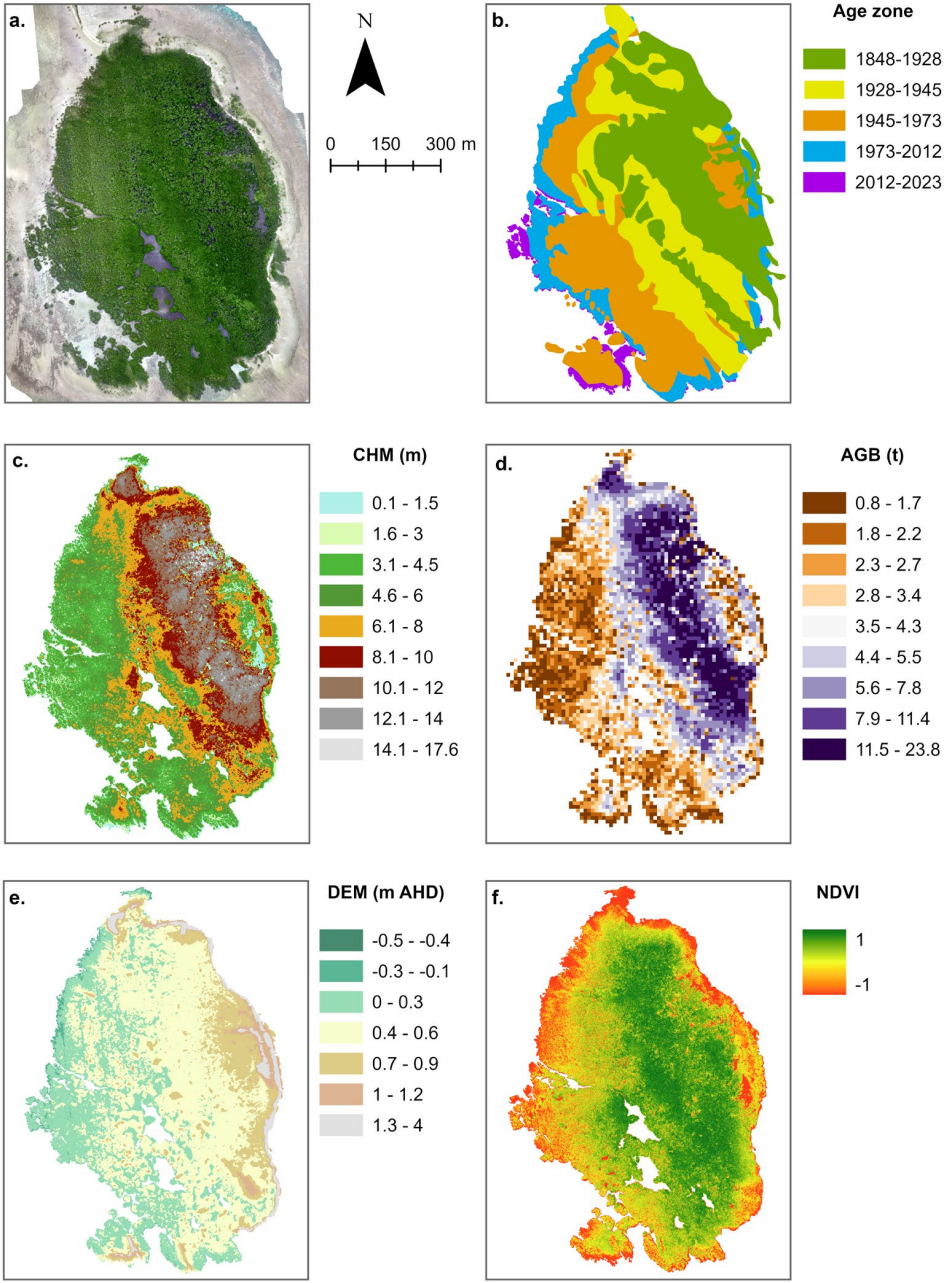
Datasets generated in this study include the 2023 orthomosaic (a), age zones (b), canopy height model (CHM) (c), above‐ground biomass model (AGB) (10 m resolution) (d), digital elevation model (DEM) (0.25 m resolution) (e), and Normalized Difference Vegetation Index (NDVI) (0.1 m resolution) (f).

**FIGURE 6:**
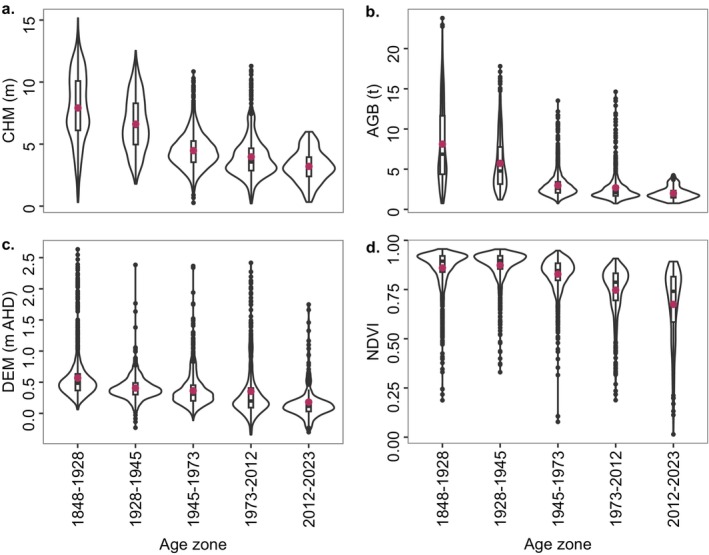
Distribution of canopy height from the Cal‐CHM (a), biomass from the AGB model (b), surface elevation from the DEM (c), and NDVI (d) across age zones. Values were extracted from rasters at 10 m resolution.

There is a general pattern of increasing canopy height (i.e., CHM), biomass (i.e., AGB), surface elevation/tidal position (i.e., DEM), and canopy greenness (i.e., NDVI) with increasing forest age; however, NDVI decreased between the 1928–1945 and 1848–1928 age zones (Figures 5 and 6). Canopy height ranged between 0.21 and 15.16 m with a mean ± SE of 5.97 ± 0.04 m (Figure 6a). Biomass ranged between 0.74 and 23.79 t per pixel (between 73.5 and 2378.7 t ha^−1^) with a mean ± SE of 5.19 ± 0.03 t (519.7 ± 3.11 t ha^−1^) (Figure 6b). The mean AGB per hectare and total AGB of the forest were 519.7 t ha^−1^ and 22,974 t, respectively. Mangrove forest surface elevations ranged between −0.34 and 2.63 m AHD with a mean ± SE of 0.43 ± 0.005 m AHD (Figure 6c). Greenness values ranged between 0.01 and 0.96 with a mean ± SE of 0.83 ± 0.002 (Figure 6d).

Distribution of raster values in each age zone is generally unimodal; however, there is a broad bimodal distribution of canopy height and biomass in the two oldest age zones (i.e., pre‐1945) (Figure 6). ANOVA indicated significant variation in the CHM, AGB, DEM, and NDVI across age zones (*p* < 0.0001) (Table A7). Additional tests using Tukey HSD indicated significant differences in raster values between most age zones, with a few exceptions detailed in the Appendix, Table A7. The mixed‐model analysis indicated age had a significant effect on AGB (*p* < 0.0001), whereas tidal position (i.e., DEM) did not have a significant effect on AGB (*p* = 0.7944). The combined effect of age and tidal position on AGB was significant (*p* < 0.0001).

**FIGURE 7:**
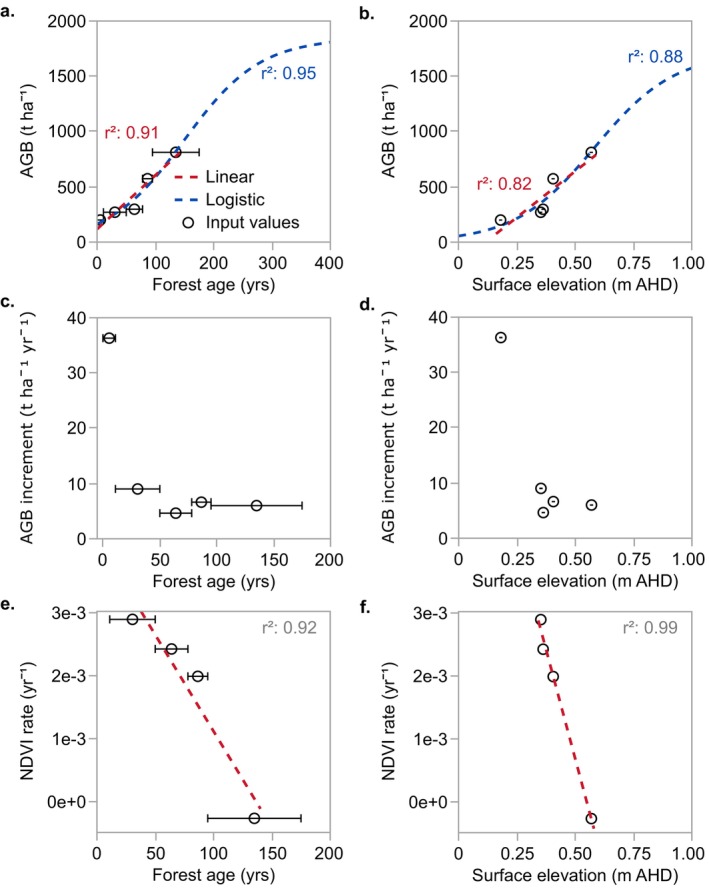
Mangrove biomass accumulation using linear and logistic regressions with age (a) and surface elevation (i.e., tidal position) (b). AGB values are summed for each age zone. Regression parameters and *r* ^2^ values are provided in Table A6. Mangrove production with age and surface elevation for total AGB production indicated by annual AGB increments (c, d) and canopy production indicated by NDVI rate (e, f). Age error bars are ± the mid‐point of age zones, and surface elevation error bars are ± SE of the mean DEM values but were too small to plot in b, d, and f. Note that only four data points for NDVI are provided, as the rate is calculated between the adjacent age zones.

The total AGB per hectare in each age zone increased linearly with age and surface elevation (i.e., tidal position) but was also explained by a logistic regression, where AGB asymptotes with age (Figure 7a) and tidal position (Figure 7b). The mean ± SE of biomass within age zones ranged from 199.7 ± 6.19 t ha^−1^ in the youngest forest to 812.0 ± 12.9 t ha^−1^ in the oldest forest.

Mangrove total AGB production and canopy production exhibited different patterns with age and tidal position, where total AGB production declined rapidly in the youngest two age zones, and canopy production remained high until declining into the oldest forest (Figure 7c–f). The mean ± SE of the mean annual AGB increment was 12.5 ± 5.4 t ha^−1^ yr.^−1^, peaking at 36.3 t ha^−1^ yr.^−1^ in the 2012–2023 age zone, positioned at 0.18 ± 0.02 m AHD, followed by relatively steady rates after 11 years between 9.02 and 4.64 t ha^−1^ yr.^−1^, positioned between 0.35 ± 0.02 and 0.57 ± 0.01 m AHD (Figure 7c,d). NDVI production rate declined linearly with age and tidal position and was greatest in younger forests aged < 50 years positioned between 0.18 ± 0.02 and 0.35 ± 0.02 m AHD (Figure 7e,f). NDVI declined rapidly into a negative production rate in the oldest two zones between 135 ± 40 and 86.5 ± 8.5 years old, positioned between 0.57 ± 0.01 and 0.41 ± 0.01 m AHD.

## 4 Discussion

The use of an RPA‐derived ultra‐high‐resolution LiDAR point cloud to generate elevation models calibrated using RTX‐GPS created an accurate DEM, DSM, and CHM, facilitating extrapolation of biomass across the mangrove forest. Timeseries mapping extending back to 1928 provided the basis for generating relationships between the high accuracy datasets (i.e., DEM, CHM, AGB, and NDVI) and forest age. Together, the dense point cloud, collection of field data across different mangrove age zones and structures, range of tree heights characterized in the CHM, and application of a region‐ and species‐specific allometric equation provide a high degree of confidence in biomass estimates. Moreover, total tree AGB at Low Isles was within the range of values for various stem diameters using allometric equations for *R. stylosa* in eastern and western Australia (Clough, Dixon, and Dalhaus 1997; Clough and Scott 1989), confirming the values produced in this study. The approach of extrapolating biomass from plots to the entire mangrove forest extent using high‐density LiDAR data has been applied elsewhere, improving biomass model accuracy (Salum et al. 2021; Wang et al. 2020). The biomass model in this study was representative of ~90% of the CHM data (Figure A3), despite logistical constraints causing data gaps in plots, further confounded by low tree density in the oldest forest. The biomass data beyond the range of the CHM are concentrated in the oldest forest (i.e., heights > 10.1 m). Therefore, we have reduced confidence in the biomass estimates for the oldest forest, where the trees are the tallest and exhibit the greatest structural complexity (e.g., leaning trees).

The average LiDAR‐derived AGB at Low Isles (519.7 ± 3.11 t ha^−1^) was greater than reported for the Howick Group of low‐wooded islands (i.e., coral reef setting) dominated by *R. stylosa* further north on the Great Barrier Reef (92.8–326.4 t ha^−1^) (Hamylton et al. 2023), and for *R. stylosa* at the West Alligator River (329.1 t ha^−1^) and Darwin Harbour (178.5 t ha^−1^) (Salum et al. 2021). Greater biomass of *R. stylosa* at Low Isles suggests climatic and edaphic factors and sediment dynamics are favorable for dense biomass accumulation. However, the mean biomass production rates at Low Isles (12.5 ± 5.4 t ha^−1^ yr.^−1^) were lower than reported for *R. stylosa* in Indonesia (50.1 t ha^−1^ yr.^−1^) (Analuddin et al. 2020) and *R. apiculata* and *R. stylosa* forests ~15 km from Low Isles in the Daintree River (25.9 t ha yr.^−1^) (Clough 1992). Mangroves appear to be slower growing at Low Isles but reach high biomass stock over time. However, biomass production rates may be slower being measured across broad age ranges and thereby not reflective of more recent production (Saenger 2002).

High biomass stock at Low Isles may correspond to variation in rainfall, as this is known to enhance mangrove biomass across Australia and the globe (Bucher and Saenger 1994; Simard et al. 2019). Higher mean annual rainfall characterizes the southern regions of the northern Great Barrier Reef, exceeding 2000 mm at Low Isles compared to between 1460 and 1773 mm near the Howick Group, West Alligator River, and Darwin Harbour (Bureau of Meteorology 2024). Moreover, comparable *R. stylosa* AGB (562 t ha^−1^) was reported in southeast Sulawesi, Indonesia, a region where annual rainfall exceeds 3000 mm yr.^−1^ (Analuddin et al. 2020). However, AGB of *R. apiculata* and *R. stylosa* on islands of the Torres Strait where mean annual rainfall is ~1400–1800 mm was comparable to Low Isles, although varied considerably ranging between 478.2 and 702.0 t ha^−1^ (for trees 12.0–18.1 m tall) and between 159.0 and 487.5 t ha^−1^ (for trees 8.3–19.0 m tall), respectively (Duke et al. 2015). Although Low Isles and the Torres Strait Islands have similar rainfall totals, the distribution of rainfall throughout the year may differ because of a strong influence of monsoonal climates at Low Isles in summer, thereby contributing to biomass accumulation.

Tree density or basal area may be influencing the high biomass at Low Isles, despite individual mangroves being < 15 m tall (short relative to mangroves elsewhere). Mangroves at Low Isles were generally taller (mean plot heights between 2 and 10 m), had higher mean basal area (53 m^2^ ha^−1^, Table A5), but similar stem density (4583 stems ha^−1^, Table A5) compared to mangroves of the Howick Group. Mangroves of the Howick Group had shorter mean tree heights (1–8 m), smaller mean tree basal area (39 m^2^ ha^−1^) and similar mean stem density (4553 stems ha^−1^) to Low Isles mangroves (Hamylton et al. 2023). Evidently, the taller trees and greater trunk diameter underpin greater biomass in the present study relative to previous work on similar low‐wooded islands (Hamylton et al. 2023). Other conditions such as soil structure, hydrology, reduced anthropogenic disturbance, suitable salinity, and nutrient availability may also explain the comparatively higher mangrove biomass at Low Isles (Clough 1992; Hayes et al. 2017; Saenger and Snedaker 1993; Saintilan 1997).

### 4.1 Mangrove Biomass Is Related to Forest Age, but Influenced by Intertidal Position

Biomass was related to forest age, whereby the westerly expansion of mangroves across the reef platform over the past ~90 years encouraged increasing canopy height, basal area, and total AGB with age. Mangrove shoreline progradation to lower intertidal positions means that greater biomass is found at landward and higher intertidal positions, corresponding to the oldest forest. This aligns with other studies showing mangrove AGB increases with age (Estrada and Soares 2017; Nguyen et al. 2004; Ren et al. 2010), which is expected as biomass accumulates incrementally over time. As mangroves mature, AGB accumulates primarily by additions to basal area and height (i.e., woody components), causing larger biomass stock in older forests, as observed at Low Isles and in other studies (Alongi 2002; Alongi and Zimmer 2024; Lovelock et al. 2010). Incremental increases in basal area and height encouraged biomass accumulation despite declines in stand density with age, and this process of self‐thinning as stands mature is ascribed to competition for light and other resources (Duke 2001; Fromard et al. 1998; Westoby 1984). Similar to Low Isles, patterns of increasing height and biomass were related to age and stand density along a 70‐year mangrove sequence in French Guiana, with greater biomass associated with older, sparser stands further from the prograding shoreline (Fromard et al. 1998; Walcker et al. 2018). However, this trend is not representative of all prograding shorelines, with complex structural variation across age zones on a seaward prograding mangrove shoreline in New Zealand attributed to pulse phases of establishment and recruitment of younger mangroves in the older forest (Lovelock et al. 2010). As such, a relatively uninterrupted pattern of mangrove establishment and growth possibly occurred at Low Isles, with a cohort of mangroves of similar age still dominating each zone.

Mangrove biomass was related to tidal position; however, this pattern emerged because of age and tidal position being positively related and a pronounced influence of age on biomass. We note that some correlation between age and tidal position cannot be discounted, which may affect the interpretation of their independent contributions. Nevertheless, tidal position alone was not a significant control of biomass, but when combined with age, it had a significant effect on biomass and implies collinearity between these variables. These associations arise as total biomass is the product of structural development and biomass production over time, influenced by inundation regimes (Alongi 2002; Lara and Cohen 2006; Sherman et al. 2003). Sediment accumulation and surface elevation have developed over time, concurrently with increases in tree height and biomass as the forest ages at Low Isles. Complex feedback between these processes may have influenced the spatio‐temporal patterns of biomass accumulation across the tidal frame. Moreover, the observed relationship between biomass and tidal position is related to the prograding shoreline, and in settings where shorelines are stable and lateral extension is landward or negligible, biomass may be negatively related to tidal position (Nguyen et al. 2004).

### 4.2 Mangrove Productivity Is Related to Forest Age and Tidal Position

Biomass production, measured using mean annual increments of AGB, generally declined with age and tidal position. This contrasts with the relationship with biomass stock, which is a cumulative measurement of production. Accordingly, the location of peak biomass production does not correspond to where biomass stock is the greatest. Biomass production peaked in the youngest forest < 11 years old at the lowest intertidal position and then steadied at relatively low rates in zones greater than 30.5 ± 19.5 years old. Patterns of peak production in the younger forest at Low Isles align with evidence of rapid growth and biomass accumulation in younger mangroves and declines in biomass productivity with increasing age (Gong and Ong 1990; Lovelock et al. 2010; Ong 1993; Ren et al. 2010).

Both age and tidal position were optimal for mangrove growth in the youngest forest at the lowest intertidal position at Low Isles, and links between inundation regimes and optimal conditions for plant growth have been proposed in previous work (Hutchings and Saenger 1987; Krauss et al. 2006; Sherman et al. 2003). As such, it is expected that biomass addition is low when mangrove propagules first establish but increases and peaks in the following stage in the youngest forest at Low Isles, aligning with optimal tidal positions for *R. stylosa* growth on the reef platform. A similar pattern was observed elsewhere, showing incremental mangrove biomass addition peaked along a seaward‐landward inundation gradient corresponding to favorable environmental conditions for growth (Suwa et al. 2008). The location of peak biomass production will align with the zone of optimal conditions and may vary across different sites depending on the tolerance of species to inundation and the elevation of establishment with reference to the expanding front.

Canopy production, indicated by NDVI change between age zones, remained high into forests aged between 70 and 100 years and across most intertidal positions of the forest; thereafter, it declined in the oldest forest. Higher canopy production rates across a broad range of forest ages at Low Isles align with the increasing photosynthetic production of *R. apiculata* with age in Southeast Asia, peaking between 25 and 30 years and approaching an asymptote by ~40 years (Alongi 2002). Photosynthetic production appears to remain high across forest ages and lower tidal positions at Low Isles, only diminishing once mangrove health and structure decline. Declining canopy production in the oldest forest may be linked to enhanced defoliation associated with exposure to prevailing winds and cyclones on the windward margin of Low Isles, where the tallest trees are located, and deteriorating health and leaning of old trees were observed (personal obs.). Although direct linkages between declining canopy production and cyclones were not quantified here, the impacts of cyclones on *R. stylosa* structure have been examined in nearby locations, with taller trees being more susceptible to damage by extreme wind speeds (Asbridge et al. 2018). Additionally, canopy production may be influenced by self‐thinning as mangroves mature (Duke 2001; Fromard et al. 1998) and was evident by declining stand density with age and broader distribution of canopy height and biomass in older stands at Low Isles. Senescence and mortality associated with mangrove aging into the later stages of maturity (Duke 2001; Fromard et al. 2004) may also be starting to occur at Low Isles, influencing canopy productivity.

Spatial patterns of canopy production contrasted with biomass production at Low Isles, representing different aspects of mangrove productivity. Canopy production exhibited a broader tolerance range on the basis of age and tidal position and appeared to be more strongly influenced by exposure to damaging conditions and declining tree density. In contrast, age and tidal position appeared to have a greater influence on biomass production. Optimal conditions for biomass production are apparent at lower tidal positions, whereas optimal conditions for canopy production appear to remain across a broader range of intertidal positions, albeit still relatively narrow given the small vertical range of the *R. stylosa* forest at Low Isles. Contrasting patterns in canopy and biomass production may also be because of shifts in the allocation of biomass from photosynthetic production, where varying amounts of carbon are allocated to AGB, lost as litterfall or respiration, or allocated to below‐ground root production (Alongi 2002; Clough, Ong, and Gong 1997).

As mangroves mature, canopy height approaches a maximum and biomass production rates diminish (Fromard et al. 1998; Suwa et al. 2008), at which point a logistic regression more appropriately represents biomass accumulation with age. The linear trajectory of biomass accumulation with age and tidal position at Low Isles suggests the oldest forest, aged 135 ± 40 years, has not yet reached the later stages of maturity. Despite this, declining biomass production with continued aging and increasing tidal position was predicted using a logistic regression, causing biomass to asymptote. Maximum biomass was predicted at > 1500 t ha^−1^, although the linear trajectory of biomass accumulation throughout forest growth at Low Isles and the lack of precision in forest age data imply substantial variation in the possible maximum biomass value. This value is also much higher than predicted for natural and reforested mangroves across the globe, of < 800 t ha^−1^ (Lovelock et al. 2022). However, it may be realistic, where reliable field‐based plot data at Low Isles indicated biomass exceeding 1000 t ha^−1^ in the older zones (Figure 3d). Nevertheless, slow biomass production rates in older forest zones and structural decline and senescence in the oldest forest indicate that mangroves are approaching later stages of maturity and equilibrium biomass at Low Isles.

Mangroves at Low Isles appear to be approaching maturity at a slower rate than other natural mangrove stands and plantations, approaching a biomass asymptote between ~20 and 40 years old (Adame et al. 2018; Lovelock et al. 2022; Phan et al. 2019; Sidik et al. 2019). This implies conditions influencing biomass accumulation at Low Isles have remained stable for longer. However, mangrove growth curves compiled from a broader range of chronosequence studies indicate carbon storage increases until at least 80–100 years, despite declining production rates (Alongi and Zimmer 2024), aligning with continued biomass addition into the oldest forest at Low Isles. Older estimates of biomass equilibrium at Low Isles and by Alongi and Zimmer (2024) may relate to the older age of stands investigated and greater consideration of natural mangrove stands. Moreover, rapid biomass accumulation and an earlier approach to maturity of mangrove plantations in Southeast Asia could reflect mangrove seedling planting at optimal tidal positions and in favorable tropical climates for growth (Saenger 2002), with production declining in relatively younger mangroves as tidal positions become suboptimal for growth. This process is influenced by organic and inorganic sediment accumulation, which influences substrate position and varies across the tidal frame (Rogers et al. 2022), thereby influencing inundation and production (Sherman et al. 2003). Therefore, temporal variation in biomass accumulation patterns across different settings may also be influenced by variation in sediment accumulation processes influencing rates of surface elevation change.

### 4.3 Conceptualizing the Influence of Age and Tidal Position on Mangrove Forest Development

**FIGURE 8:**
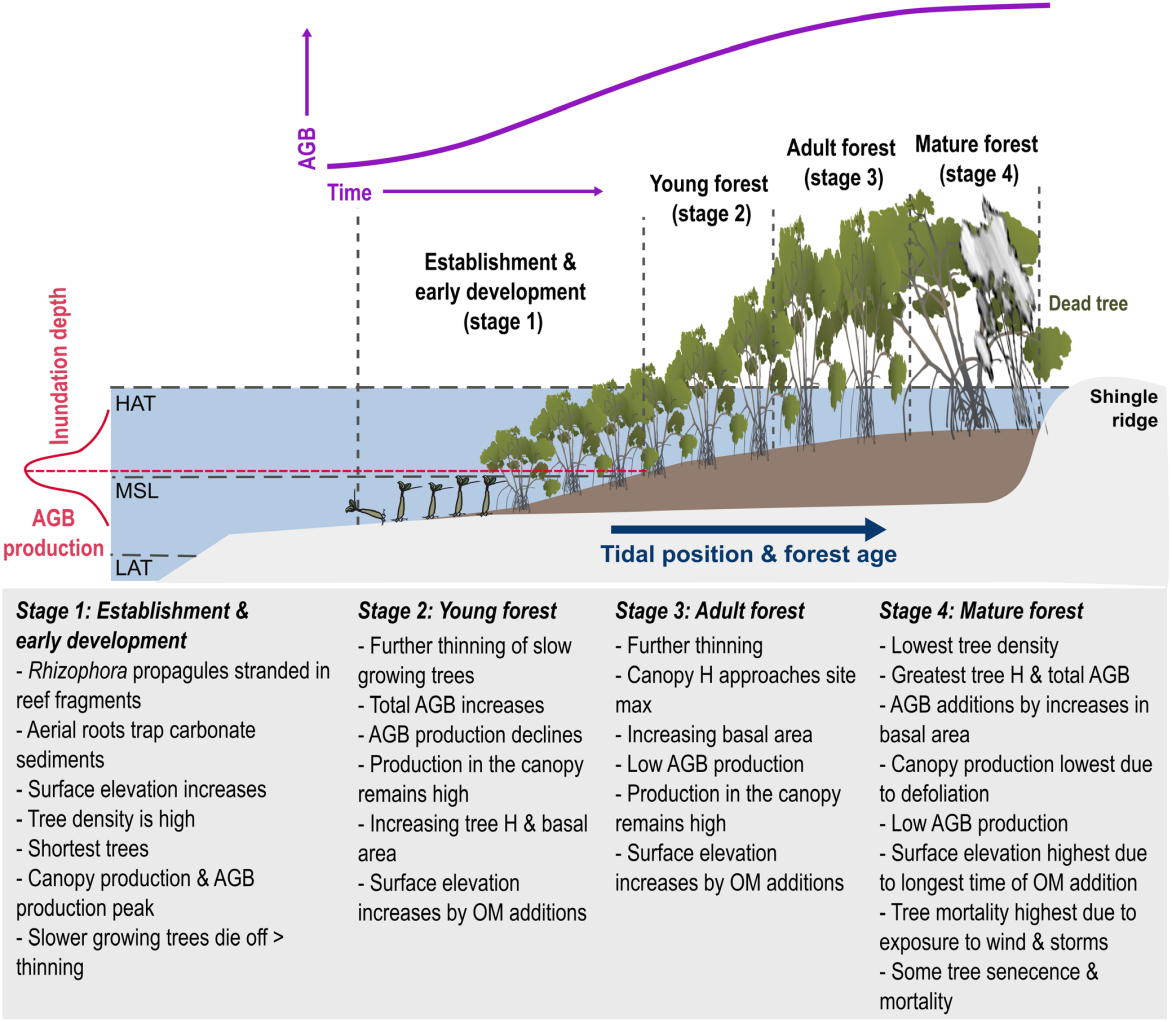
Conceptual model of mangrove forest development at Low Isles, Great Barrier Reef.

The dynamics of mangrove establishment, development, and substrate evolution at Low Isles are conceptualized in Figure 8 across four stages. The first stage of establishment and early development is characterized by a pattern of mangrove expansion across the exposed reef platform, facilitated by mangrove propagule establishment and development. On the protected western margin of the reef platform, mangrove establishment may be influenced by windows of opportunity, whereby periodic shifts in hydrodynamics and sediment dynamics provide suitable conditions for opportunistic establishment (Balke et al. 2011, 2015). Once established, the development of *Rhizophora'*s aerial root systems traps sediment, and thereafter initiates a positive feedback cycle whereby mangrove structural development is accompanied by progressive increases in substrate elevation as below‐ground organic matter accumulates (Marchand et al. 2003). The latter portion of this stage is marked by the early development of young mangroves, with tree density initially high but declining because of competition, and canopy production peaking, and addition of tree height and therefore AGB production peaking (i.e., 2012–2023 zone, Figure 6, Table A5).

The second stage in the young forest is marked by declines in tree density as taller trees outcompete shorter trees for resources (i.e., light). The young forest, with ages ranging from 1973 to 2012, exhibits greater canopy complexity (Figure 6), and self‐thinning is evident from plot data, as has been shown elsewhere (Fromard et al. 1998; Westoby 1984). Despite evidence of self‐thinning, canopy productivity remains high and AGB and tree height continue to increase, but AGB production declines.

Transitioning into an adult forest in the third stage, the upper canopy closes, indicated by the highest mean NDVI in the 1928–1945 zone. Here, trees are approaching the site maximum tree height, while self‐thinning continues. Field plots 4A–C were estimated to have been established since 1973 and are characteristic of a closed mature forest (Duke 2001), whereby trees are slender, tall, and dense and have no undercanopy saplings and young trees (Figure 3, Table A5). Canopy production remains high, AGB production remains low, but tree height and AGB continue to increase in the adult forest.

In the fourth stage, mangroves develop into a mature forest at the site's maximum canopy height, and the oldest forest (i.e., 1848–1928) is in the early stages of maturity, indicated by increased canopy thinning and basal thickening in plots 1A–D and 2A–D (Figure 3, Table A5). However, regression analyses between biomass accumulation and forest age suggest mangroves have not yet reached maturity but will approach the later stages of maturity, influenced by senescence with increasing age and tidal position. Here, the relationship of increasing canopy height, AGB, and tidal position becomes more complex. As such, canopy height and AGB in the older zones show signs of greater complexity with bimodal distribution (Figure 6). However, the distribution of lower heights may be related to the leaning of old trees on the windward side of the island. Tree height and AGB are at the site's maximum at this stage, and AGB production remains low, and canopy production declines substantially. Reduced canopy density in the 1848–1928 zone may cause the evident decrease in NDVI because of a reduction in leaf area. According to previous models, this zone would represent the mangrove “cemetery,” where mature trees reach senescence, and mangrove stands initiate turnover cycles with younger trees replacing old trees (Duke 2001; Fromard et al. 2004). However, this may also be influenced by exposure to prevailing winds and storms in this zone at Low Isles.

## 5 Conclusion

Mangrove forest development at Low Isles influenced positive associations between forest age and tidal position and a relationship between biomass accumulation and forest age. Biomass production declined with forest age and as tidal positions increased, but biomass continued to accumulate into the oldest forest aged 135 ± 40 years. The mangrove forest at Low Isles stores more than 10,500 t of carbon or ~240 t C ha^−1^ in its AGB, on the basis of a conversion factor of 0.459 for a similar species (Kauffman et al. 2011). Mangrove carbon stores have accumulated over the past ~150 years, particularly during the accelerated periods of expansion since 1928. Providing environmental conditions remain suitable for mangrove survival and expansion, increases in carbon stock are expected with continued progradation of mangroves across the reef platform. However, if processes of sediment accumulation are insufficient to maintain substrate position under conditions of relative sea‐level rise, patterns of expansion and biomass accumulation may change. Moreover, if cyclones intensify as predicted, this could affect mangrove senescence and their capacity to recover, influencing biomass accumulation. If above‐ and below‐ground production are synchronized, it is expected that peak below‐ground production occurs throughout early development into the young forest stage. Although information about below‐ground substrate development of the mangrove forest is required to project the longer‐term additionality of carbon to AGB under conditions of accelerating sea‐level rise. Moreover, it is anticipated that structural complexity will increase with the oldest forest entering later stages of maturity and will have implications for carbon storage at Low Isles.

## Author Contributions

**Brooke M. Conroy:** conceptualization (equal), data curation (lead), formal analysis (lead), investigation (lead), methodology (equal), validation (lead), visualization (lead), writing – original draft (lead), writing – review and editing (lead). **Sarah M. Hamylton:** conceptualization (supporting), data curation (supporting), formal analysis (supporting), funding acquisition (supporting), investigation (supporting), methodology (supporting), resources (supporting), supervision (supporting), writing – review and editing (supporting). **Jeffrey J. Kelleway:** conceptualization (supporting), investigation (supporting), methodology (supporting), supervision (supporting), writing – review and editing (supporting). **Emma F. Asbridge:** investigation (supporting), methodology (supporting), writing – review and editing (supporting). **Colin D. Woodroffe:** conceptualization (supporting), writing – review and editing (supporting). **Kerrylee Rogers:** conceptualization (equal), formal analysis (supporting), funding acquisition (lead), investigation (supporting), methodology (supporting), project administration (lead), resources (supporting), supervision (lead), validation (supporting), writing – review and editing (supporting).

## Conflicts of Interest

The authors declare no conflicts of interest.

## Data Availability

Field plot data are provided in Table A5 of the Appendix. Spatial datasets and field plot individual tree data can be accessed at: https://doi.org/10.5061/dryad.5tb2rbpfg.

## Appendix A

### Supplementary Materials

**TABLE A1:** ANOVA and Tukey HSD test results for field data subplots for DBH.

	Plot 1A–D	Plot 2A–D	Plot 4A–D
**ANOVA**			
Df	3	3	3
Sum_Sq	290.8	195.1	481.8
Mean_Sq	96.9	3005.5	160.6
*F*_value	2.6	0.7	2.2
Pr(> *F*)	0.0577	0.5379	0.0896
**Tukey HSD tests *p* value for comparison of subplots**			
B–A	0.5614	0.7787	0.3342
C–A	0.8462	0.5691	0.9114
D–A	0.5215	0.5421	0.4893
C–B	0.2724	0.9213	0.1464
D–B	0.0476	0.8921	0.9792
D–C	0.9943	0.9999	0.2232

**TABLE A2:** Number of measurements for each field plot for tree height and DBH.

Plot	Height	DBH
1A	0	19
1B	0	19
1C	0	8
1D	0	18
2A	0	2
2B	0	11
2C	0	12
2D	0	13
3	75	75
4A	37	40
4B	0	21
4C	0	28
4D	0	30
5A	64	64
5B	35	35
6	133	133
7	75	75
8	222	222

**TABLE A3:** RPA survey details.

	RPA‐multispectral	RPA‐LiDAR and optical
Date	June 2022	July 2023
Drone, sensor	Phantom 4, MicaSense RedEdge‐M sensor	DJI Matrice 300 RTK, Zenmuse L1, optical sensor
Flight speed (m s^−1^)		5
Altitude (m)	120	60
Conditions	Fine‐cloudy, winds < 15 km h^−1^, low tide	Fine‐cloudy, winds < 15 km h^−1^, low tide
Point cloud details		566.9 pts m^−2^ (spacing 0.042), 611,195,800 points, (16.4% ground, 83.6% undefined)
Optical image details	Red, green, blue, near‐infrared, red edge, 505 images per band (8 cm/pixel)	Red, green, blue, 970 images, (2.78 cm/pixel)

**TABLE A4:** DEM and DSM processing parameters.

	DEM processing parameters	DSM processing parameters
LAS filter	Ground	All points
Interpolation type	Binning	Binning
Cell assignment	Average	Maximum
Void fill method	Natural neighbour	Linear
Cell size (m)	0.25	0.25

**TABLE A5:** Field plot (100 m^2^) summary statistics.

Plot ID	DEM value (m AHD)		Estimated age zone	Tree density (stems ha^−1^)	Shrub density < 1.3 m (stems ha^−1^)	Tree DBH (cm)		Plot basal area (m^2^ ha^−1^)	Tree H (cm)			Tree AGB (kg)	
	Mean	SE				Mean	SE		Mean	SE	Max	Mean	SE
1A	0.59	0.002	1848–1928	1900	0	20.15	1.43	66.12				402.76	72.46
1B	0.57	0.002	1848–1928	1900	0	17.58	1.21	50.07				275.03	44.26
1C	0.59	0.003	1848–1928	800	0	22.24	2.70	34.30				534.73	143.92
1D	0.62	0.002	1848–1928	1800	0	22.89	1.39	78.73				536.53	84.37
2A	0.56	0.001	1848–1928	200	0	32.15	1.43	16.27				1173.55	139.81
2B	0.62	0.003	1848–1928	1100	0	25.29	3.77	67.56				959.74	444.67
2C	0.65	0.002	1848–1928	1200	0	22.81	1.65	51.86				525.50	96.83
2D	0.69	0.002	1848–1928	1300	100	22.55	2.63	60.39				627.24	189.58
3	0.47	0.002	1928–1945 or 1945–1973	7500	200	5.86	0.67	39.72	4.05	0.13	6.40	53.16	22.78
4A	0.49	0.007	1945–1973 or 1973–2012	4000	0	14.52	1.47	92.71	10.07	0.33	> 12	292.15	100.29
4B	0.60	0.005	1945–1973 or 1973–2012	2100	0	18.39	1.91	67.80				394.16	98.27
4C	0.45	0.002	1945–1973 or 1973–2012	2800	0	13.13	1.47	50.73				196.17	59.98
4D	0.65	0.003	1945–1973 or 1973–2012	3000	0	17.43	1.43	85.59				330.56	69.55
5A	0.12	0.004	1945–1973	6400	0	9.36	0.87	67.84	7.29	0.36	11.00	110.66	40.53
5B	0.39	0.000	1945–1973	3500	0	11.02	1.32	49.76	7.73	0.42	10.50	156.90	57.10
6	0.22	0.001	1973–2012	13300	0	4.62	0.26	31.31	4.32	0.12	7.00	13.20	2.12
7	0.08	0.002	1973–2012	7500	17500	3.61	0.35	12.91	3.16	0.15	5.80	9.47	2.24
8	0.21	0.002	1973–2012	22200	9700	2.12	0.19	21.61	2.31	0.06	4.90	7.50	3.14

**TABLE A6:** Logistic and linear regression parameters.

Logistic	Growth rate ± SE	Inflection point ± SE	Asymptote ± SE	*r* ^2^
Field plot mean tree H (m) vs field plot AGB (*t*)	0.323 ± 0.194	11.8 ± 11.3	32.1 ± 69.6	0.91
Forest age (years) vs LiDAR AGB (*t* ha^−1^)	0.015 ± 0.011	149.3 ± 211.6	1841.6 ± 3246.9	0.95
Surface elevation (m AHD) vs LiDAR AGB (*t* ha^−1^)	5.83 ± 7.03	0.58 ± 0.81	1713.6 ± 4188.7	0.88

Linear	Intercept ± SE	Slope ± SE	*r* ^2^
Forest age (years) vs LiDAR AGB (*t* ha^−1^)	116.99 ± 68.49	4.87 ± 0.87	0.91
Surface elevation (m AHD) vs LiDAR AGB (*t* ha^−1^)	−193.88 ± 180.06	1667.5 ± 456.53	0.82

**TABLE A7:** ANOVA and Tukey HSD test results for each raster against forest age using the mid‐point of the age zones.

	CHM	AGB	DEM	NDVI
**ANOVA**				
Df	4	4	4	4
Sum_Sq	11711.8	23356	39.0	9.8
Mean_Sq	2928.0	5839	9.7	2.5
*F*_value	645.0	530.1	107.0	311.8
Pr(> *F*)	< 0.0001	< 0.0001	< 0.0001	< 0.0001
**Tukey HSD tests *p* adj for each age zone**				
1959–1936.5	< 0.0001	< 0.0001	0.054	< 0.0001
1992.5–1936.5	< 0.0001	< 0.0001	< 0.05	< 0.0001
2017.5–1936.5	< 0.0001	< 0.0001	< 0.0001	< 0.0001
1888–1936.5	< 0.0001	< 0.0001	< 0.0001	0.199
1992.5–1959	< 0.0001	0.477	0.908	< 0.0001
2017.5–1959	< 0.0001	< 0.05	< 0.0001	< 0.0001
1888–1959	< 0.0001	< 0.0001	< 0.0001	< 0.0001
2017.5–1992.5	< 0.05	0.245	< 0.0001	< 0.0001
1888–1992.5	< 0.0001	< 0.0001	< 0.0001	< 0.0001
1888–2017.5	< 0.0001	< 0.0001	< 0.0001	< 0.0001
